# Ecogeography of teosinte

**DOI:** 10.1371/journal.pone.0192676

**Published:** 2018-02-16

**Authors:** José de Jesús Sánchez González, José Ariel Ruiz Corral, Guillermo Medina García, Gabriela Ramírez Ojeda, Lino De la Cruz Larios, James Brendan Holland, Roberto Miranda Medrano, Giovanni Emmanuel García Romero

**Affiliations:** 1 Universidad de Guadalajara, Centro Universitario de Ciencias Biológicas y Agropecuarias, Zapopan, Jalisco, Mexico; 2 Instituto Nacional de Investigaciones Forestales Agrícolas y Pecuarias, Centro de Investigación Regional del Pacífico Centro, Campo Experimental Centro Altos de Jalisco, Guadalajara, Jalisco, Mexico; 3 Instituto Nacional de Investigaciones Forestales Agrícolas y Pecuarias, Centro de Investigación Regional del Norte Centro, Campo Experimental Zacatecas, Calera, Zacatecas, Mexico; 4 USDA-ARS Plant Science Research Unit, Department of Crop and Soil Sciences, North Carolina State University, Raleigh, North Carolina, United States of America; New York State Museum, UNITED STATES

## Abstract

Adaptation of crops to climate change has motivated an increasing interest in the potential value of novel traits from wild species; maize wild relatives, the teosintes, harbor traits that may be useful to maize breeding. To study the ecogeographic distribution of teosinte we constructed a robust database of 2363 teosinte occurrences from published sources for the period 1842–2016. A geographical information system integrating 216 environmental variables was created for Mexico and Central America and was used to characterize the environment of each teosinte occurrence site. The natural geographic distribution of teosinte extends from the Western Sierra Madre of the State of Chihuahua, Mexico to the Pacific coast of Nicaragua and Costa Rica, including practically the entire western part of Mesoamerica. The Mexican annuals *Zea mays* ssp. *parviglumis* and *Zea mays* ssp. *mexicana* show a wide distribution in Mexico, while *Zea diploperennis*, *Zea luxurians*, *Zea perennis*, *Zea mays* ssp. *huehuetenangensis*, *Zea vespertilio* and *Zea nicaraguensis* had more restricted and distinct ranges, representing less than 20% of the total occurrences. Only 11.2% of teosinte populations are found in Protected Natural Areas in Mexico and Central America. Ecogeographical analysis showed that teosinte can cope with extreme levels of precipitation and temperatures during growing season. Modelling teosinte geographic distribution demonstrated congruence between actual and potential distributions; however, some areas with no occurrences appear to be within the range of adaptation of teosintes. Field surveys should be prioritized to such regions to accelerate the discovery of unknown populations. Potential areas for teosintes *Zea mays* ssp. *mexicana* races Chalco, Nobogame, and Durango, *Zea mays* ssp. *huehuetenangensis*, *Zea luxurians*, *Zea diploperennis* and *Zea nicaraguensis* are geographically separated; however, partial overlapping occurs between *Zea mays* ssp. *parviglumis* and *Zea perennis*, between *Zea mays* ssp. *parviglumis* and *Zea diploperennis*, and between *Zea mays* ssp. *mexicana* race Chalco and *Zea mays* ssp. *mexicana* race Central Plateau. Assessing priority of collecting for conservation showed that permanent monitoring programs and *in-situ* conservation projects with participation of local farmer communities are critically needed; *Zea mays* ssp. *mexicana* (races Durango and Nobogame), *Zea luxurians*, *Zea diploperennis*, *Zea perennis* and *Zea vespertilio* should be considered as the highest priority taxa.

## Introduction

Mexico-Central America is among the areas with the greatest wealth of flora in the world. It has been identified as the center of origin and diversity of cultivated plants that have acquired considerable importance on a global scale; Mexico is one of the four countries of the world with the highest numbers of animal and plant species [1, 2]. One of the most important characteristics of Mexico’s floral diversity is that 12% of the genera and 50–60% of its total species are endemic; that is, their distribution is restricted to Mexico. This is the case for some teosinte species (*Zea* spp.).

The wild relatives of maize, collectively referred to as teosinte, are represented by annual and perennial diploid species (2n = 20) and by a tetraploid species (2n = 40). They have been reported within the tropical and subtropical areas of Mexico, Guatemala, Costa Rica, Honduras, El Salvador and Nicaragua as isolated populations of variable dimensions occupying from less than one acre to several square kilometers. Teosinte grows in a variety of ecological conditions from hot and humid regions to temperate and dry valleys; it can be found on the edges of and within maize fields, on the edges of small streams, in open woods, on rocky slopes of mountains, and as a constituent of the herbaceous cover in grassy areas. The distribution of teosinte extends from the southern part of the Western Sierra Madre of the State of Chihuahua, Mexico to the western coast of Nicaragua, El Salvador and Costa Rica. Populations do not have a uniform distribution across the landscape; rather, they tend to be associated with specific climate, soil, and human cultural conditions.

During most of the first half of the 20^th^ century, the work by Collins in Mexico [3] and by Kempton and Popenoe [4] in Guatemala represented the most important references on the distribution of teosinte. Wellhausen *et al*. [5] in the classic book “Razas de Maiz en Mexico” showed a map of teosinte distribution in Mexico, unfortunately, there is no text accompanying the map or a guide to locate with precision the sites with teosinte presence. The systematic collection of teosinte began during the 1960’s and 1970’s by Wilkes and Kato [6, 7]. Wilkes [6] published a remarkable monograph on teosinte from Mexico and Guatemala. He traveled through Mexico and found teosinte in most of the locations where it was previously reported; in addition to his collections and monitoring trips during three decades, he prepared maps showing the occurrence sites of teosinte from southeast Honduras to northern Mexico [8, 9, 10, 11]. During the last 30 years, Sánchez and co-workers have explored and collected teosinte in most geographical regions in Mexico [12, 13, 14, 15, 16].

Teosinte has developed several physiologically distinct taxa, each of which has acquired morphological, ecological and chromosomal distinctness [6, 8]. There are two classifications for teosinte: Wilkes [6] identified geographic populations associated with different environments and described four races of teosinte for Mexico (Nobogame, Central Plateau, Chalco and Balsas) and two for Guatemala (Guatemala and Huehuetenango). Iltis and Doebley [17], Doebley and Iltis [18] and Doebley [19] proposed a hierarchical system of classification for *Zea*, based on the morphological, ecological and molecular features of the taxa. They divided *Zea* into two sections. Section *Luxuriantes* includes *Zea perennis* (Hitch.) Reeves & Mangelsdorf, *Zea diploperennis* Iltis, Doebley & Guzmán, and *Zea luxurians* (Durieu & Ascherson) Bird. We should consider the newly described *Zea vespertilio* Gómez-Laurito [20] and *Zea nicaraguensis* Iltis & Benz [21] as members of this section. Section *Zea* includes *Zea mays* L., which was divided into *Zea mays* ssp. *mexicana* (Schrader) Iltis for races Chalco, Central Plateau and Nobogame; *Zea mays* ssp. *parviglumis* Iltis & Doebley that includes race Balsas, *Zea mays* ssp. *huehuetenangensis* (Iltis & Doebley) Doebley for race Huehuetenango, and *Zea mays* L. ssp. *mays* for cultivated maize. Recently, Sánchez et al. [16], using evidence from multiple independent sources, reported three new taxa from Mexico within section *Luxuriantes* from the Mexican states Nayarit, Michoacán and Oaxaca.

Teosinte is the closest relative of maize; maize domestication occurred in Mexico approximately 10,000 years ago from the tropical annual teosinte, *Zea mays* ssp. *parviglumis* [22, 23]. Several authors suggested that introgression from teosinte influenced diversification within maize and the origin of the principal races of maize in Mexico [5, 24, 25, 26]. The need to adapt crops to changing climates and the availability of new molecular marker technologies with the potential to accelerate introgression breeding programs have motivated interest in the potential value of novel traits from wild species for crop improvement [27, 28]. Several studies have shown that some teosinte species harbor traits or genes that may be useful to crop improvement programs. *Zea luxurians* and *Zea nicaraguensis* are adapted to frequent rainfalls and possess unique flooding resistance traits such as the capacity to form root aerenchyma even under non-flooding conditions [29]. On the other hand, in the dry environment of Durango valleys, *Zea mays* ssp. *mexicana* populations seem to survive by drought escape mechanisms such as a very short vegetative growth period and probably by drought resistance genes. Nault [30] found that *Zea perennis* and *Zea diploperennis* showed resistance to several important viruses that attack maize whereas all other *Zea* species are susceptible. *Striga* spp., are menacing root parasites of significant importance in much of Africa and parts of Asia; one of the few resistant sources was found in *Zea diploperennis* [31]. Lennon et al. [32] evaluated BC_4_S_2_ near isogenic lines with introgressions from *Zea mays* ssp. *parviglumis* in a common B73 inbred background for resistance to gray leaf spot (GLS, *Cercospora zeae-maydis* and *Cercospora zeina*); six markers significantly associated with resistance to GLS from teosinte were identified and validated.

The adaptive range of species and the importance of environmental factors for adaptation can be revealed by ecogeographical characterization. Ecogeographic studies, aided by geographic information systems (GIS), can be very important for the understanding of the environmental conditions and associated biotic and abiotic factors to which plant species have adapted. Some new strategies have been developed to find adaptive traits from collection sites where selection pressures for the trait are more likely; these strategies are based on the work by Nikolai I. Vavilov, who was one of the first to recognize the importance of environmental conditions when searching for genetic resources for plant breeding programs [33]. These aspects will be key issues for agriculture to adapt to climate change. In addition, they may be very important to identify the most appropriate places for the regeneration of genetic resources and to design *in situ* conservation programs. The principal objective of this study was to use the historical occurrence data of teosinte, in its natural distribution areas, to conduct an ecogeographical analysis to measure the contribution of several ecological descriptors in determining current teosinte distributions, identify adaptation patterns of the different taxa of teosinte, estimate the potential value of teosinte in maize to breeding and develop models predicting potential geographic distributions.

## Materials and methods

### Occurrence data

In the present study, we geo-referenced herbarium specimen records, data on germplasm accessions, and archaeological records of teosinte. A database of teosinte occurrences was built from different sources for the period 1842–2016, yielding 2363 teosinte references. The data include germplasm bank accessions, herbarium specimens, reports, USDA Plant Inventories, papers, and other document types. Quality of geographical coordinates for most existing populations was verified *in situ* using a Global Positioning System (GPS). For historical sites, where populations no longer exist, 1:50,000 scale maps from the Instituto Nacional de Estadística y Geografía (INEGI), Google Earth maps, and the Geographic names database from the National Geospatial Intelligence Agency were used. Records coming from cultivated samples out of their natural distribution areas, and those lacking geographic information and site description, were removed from the database.

Passport data of seed bank accessions included those from the Universidad de Guadalajara, the Instituto Nacional de Investigaciones Forestales Agrícolas y Pecuarias (INIFAP), the International Maize and Wheat Improvement Center (CIMMYT) and the USA National Plant Germplasm System (NPGS). Online databases considered were from the Comisión Nacional para el Conocimiento y Uso de la Biodiversidad (CONABIO), the Atlas of Guatemalan Crop Wild Relatives, the Crop Wild Relative Global Occurrence Database (www.cwrdiversity.org) and the Global Biodiversity Information Facility (GBIF). Several herbaria provided online access to passport data: the University of Arizona, Tucson (ARIZ); the Botanic Garden and Botanical Museum Berlin-Dahlem, Germany (B); the U.S. National Fungus Collections, Beltsville, Maryland (BPI); the Colegio de Postgraduados, Montecillo, Mexico (CHAPA); the Herbario Nacional Colombiano, Instituto de Ciencias Naturales de la Universidad Nacional de Colombia, Bogotá, Colombia (COL); the Escuela Nacional de Ciencias Biológicas, IPN-Mexico (ENCB); the Field Museum of Natural History, Chicago, Ill. US (F); the Harvard University Herbaria, US (GH); the Herbario of the Universidad Autónoma de Zacatecas, Mexico (HUAZ); the Instituto de Ecología, A.C., Pátzcuaro, Mexico (IEB); the Royal Botanic Gardens, Kew, England (K); the National Herbarium of The Netherlands (L); the Lundell Herbarium, University of Texas, Austin, US (TEX-LL); the Herbario Nacional, Instituto de Biología, UNAM, Mexico (MEXU); the University of Michigan, Ann Arbor, MI, US (MICH); the Missouri Botanical Garden, Saint Louis, Missouri, US (MO); the New York Botanical Garden, US (NYBG); the Smithsonian Institution, Washington, D.C. (US); the University of Wisconsin, Madison, US (WIS); and the Instituto de Ecología, A.C., Xalapa, Veracruz, Mexico (XAL). Direct records of occurrence data were obtained from the Instituto de Botánica, Universidad de Guadalajara, Mexico (IBUG) and the Departamento de Ecología y Recursos Naturales, CUCSUR, Universidad de Guadalajara, Mexico (ZEA). Some errors in the taxonomic status of accessions in genebanks and herbaria specimens have been found repeatedly. Most populations collected by Wilkes, Iltis and Doebley, INIFAP and Universidad de Guadalajara were classified based on evaluation for morphological characters, isozyme polymorphisms and DNA markers. Thus, accurate taxonomic identification of the database records with no evaluation data was achieved by comparing to reference collections (INIFAP, Universidad de Guadalajara) and some selected publications [6, 17, 14, 16].

After integration of multiple data sources, quality control of the database used in this study was conducted by a very detailed review of every single record; for most cases, information of original herbarium specimens and germplasm accessions from type localities were used as reference. When comparing among sources of information we found that the Crop Wild Relative Global Occurrence Database (www.cwrdiversity.org) and the Global Biodiversity Information Facility (GBIF) misreported several of the occurrences of teosinte. Most teosinte accessions from CIMMYT (http://germinate.seedsofdiscovery.org/maize/) were misreported as *Zea perennis*. Based on information on type localities and original collections, only 114 of the 359 records reported as *Zea perennis* at GBIF should be considered valid. Once taxonomy was standardized and ecogeographic information was validated, data were cleaned and the final database used in this study includes only the corrected records. The teosinte database can be accessed at http://www.biodiversidad.gob.mx/genes/monitoreo_teocintles.html.

### Environmental data

The National Environmental Information System (NEIS) of INIFAP was updated and used to characterize the environmental conditions of the collecting sites by means of the GIS Idrisi Selva [34]. The update of this system included the incorporation of Central America climatic normals to interpolate and generate normal monthly rasters for maximum temperature, minimum temperature and precipitation.

Climatic information for Mexico and Central America corresponded to 3026 weather stations that had more than 90% of data for the periods 1961–2010 (Mexico) and 1961–2014 (Central America) (S1 Fig). For occurrences of teosinte before 1961, we assumed that climatic conditions before 1961 are well represented by climatology 1961–2014. Climatic information was inspected to find and eliminate data ‘out of range’ by using the program R-Climdex [35]. Missing data were estimated with the program CLIMGEN [36].

Calculation of climatic normals was made with dynamic tables in Microsoft Excel, and these normal values were used to feed interpolation processes with the Anusplin Method; interpolation processes were implemented by the Anusplin package [37] considering a resolution of 30” arc for the images to be generated. Once the normal monthly rasters were obtained for maximum temperature (Tx), minimum temperature (Ti) and precipitation (P), other monthly layers were generated with the GIS Idrisi Selva. These layers included mean temperature (Tm), thermal oscillation (OT), photoperiod (F), thermal sum (TS), growing-degree days (GDD, base temperature 12°C) and potential evapotranspiration (ETP), which was estimated with the Thornthwaite method (TH) adjusted to Penman-Monteith (PM) equation by using adjustment values obtained throughout calculations of regional deviations (Penman/Monteith—Thornthwaite) for the 26 agroclimatic regions of Mexico (S2 Fig).

Values to adjust to Penman-Monteith were obtained by calculating the median value for monthly differences between TH ETP and PM ETP calculations made for each weather station climatic record from the INIFAP-COFUPRO National Meteorological Monitoring Net.

NEIS updated to Mexico-Central America may be accessed as SIAMEXCA system in the link: http://www.inifapcirpac.gob.mx/siamexca.html.

Temperature, precipitation and evapotranspiration monthly rasters were subjected to processes of cell-value extractions by using the system ArcGis. Resulting data matrices were stored in Microsoft Excel worksheets, and used to calculate additional parameters, such as growing season [38], total humid months (MH, P≥ETP) and the 19 bioclimatic variables proposed by Hijmans et al. [39] and often used in species distribution modelling.

Furthermore, the variables solar radiation and relative humidity were added as monthly normals to the databases and the information system by recouping and interpolating assimilation data for the series 1984–2015, and derived from the NASA site about Climatology Resource for Agroclimatology (http://power.larc.nasa.gov/cgi-bin/cgiwrap/solar/agro.cgi?email=agroclim@larc.nasa.gov).

Finally, 216 variables were integrated to the database and geographical environmental information system, including geographical, topographical and monthly, seasonal and annual climatological parameters (Table 1).

**Table 1:** Environmental variables considered in the study.

Variable	*Period*			Total
	Monthly	Seasonal	Annual	
Longitude (degrees)				1
Latitude (degrees)				1
Altitude (m)				1
Minimum temperature (°C)	12	3	1	16
Maximum temperature (°C)	12	3	1	16
Mean temperature (°C)	12	2		14
Thermal range (°C)	12	3		15
Thermal sum (°C)	12	2	1	15
Growing season length (GS) (days)		1		1
GS initiation (Julian day)		1		1
GS finalization (Julian day)		1		1
Accumulated growing-degree days (GDD)	12	2	1	15
Photoperiod (h)	12	2	1	15
Precipitation (mm)	12	4	2	18
Potential evapotranspiration (ETP; mm)	12	2	1	15
Moisture index	12	4	3	19
Humid months (P≥ETP)		1	2	3
Solar radiation	12	2	1	15
Relative humidity	12	2	1	15
Bioclimatic variables	4	8	7	19
Total	148	43	22	216

### Statistical analysis

Differences among races and species for the various environmental variables were analyzed by one-way analysis of variance using SAS proc GLM [40] with race treated as a class variable. It should be noted that there was high collinearity within groups of variables, therefore F values among races and correlation coefficients were used to select variables for further analysis. At this stage, variable selection for clustering and classification (VSCC) technique was used; it is intended to find the variables that simultaneously minimize the ‘within-group’ variance and maximize the ‘between-group’ variance [41]. Principal components analysis was conducted to synthetically analyze ecogeographical data; using the first two principal components, a biplot graph was built and visualized with NTSyS 2.2 [42]. In addition, linear discriminant analysis was used to verify if the recorded sites of teosinte were correctly assigned to “geographic races” and species.

### Modeling potential geographical distribution

The MaxEnt (Maximum Entropy) model [43] V. 3.4.1 was used for modelling the geographical distribution of the different teosinte taxa. MaxEnt has been described as especially efficient at handling complex interactions between response variables and predictors [44, 45] and to be robust to small sample sizes [46, 45]. MaxEnt uses the principle of maximum entropy on presence-only data to estimate a set of functions that relate environmental variables and habitat suitability in order to approximate the species’ potential geographic distribution [47].

Two types of analyses were made to determine potential distribution of teosinte; one considering all occurrences of teosinte (all taxa), and a separate analysis for each taxon individually. Occurrence data were randomly partitioned into training (50%) and test (50%) data sets for the purpose of testing model statistical significance [48]. Model settings included a summarized model of 10 fold cross validation for taxa with more than 50 presence data, and a summarized model of 50 bootstrap replicates for taxa with less than 50 occurrence sites. Several regularization factors were tested [49] but values different than 1 did not prove to be better than 1.

Model performance was judged by estimating the Area Under the Curve (AUC) from receiver operating characteristic (ROC) plots [50], which was used to assess the goodness of discrimination of suitable versus unsuitable areas for teosintes (Models with AUC values > 0.7 are considered acceptable [51]), and binomial tests of omission (known areas of occurrence/predicted absence) were used to test whether or not these differences are significant at p < 0.05 [47, 52]. A presence/absence binary map was constructed for the teosintes by thresholding environmental aptitude with the method of selecting threshold that guarantees the lowest omission rate at a maximum logistic value. For example, when three methods such as “Equal Test Sensitivity and specificity”, “Minimum Training Presence” and “10^th^ percentile training presence (10PTP)” all of them offered the lowest omission rate (i.e. 0.005), but with an environmental aptitude logistic threshold of 0.317, 0.395 and 0.523, respectively; the 10PTP method was chosen to generate the presence/absence binary map in order to avoid overestimation of potential distribution areas. This criterion was adopted because the primary objective was to represent the most likely teosinte occurrence sites while avoiding sampling bias due to outlying occurrence records and avoiding overestimation of potential distribution areas. This criterion is appropriate assuming that the sampled teosinte occurrences are very close to the true distribution and that errors in geo-references were minimized by data curation to avoid overestimation of potential distribution areas [53, 54]. The Jackknife analysis tool provided by MaxEnt was used to identify the most important variable influencing the final teosinte distribution models [47].

### Ecological descriptors

Since some taxa had only a few occurrences in the data, MaxEnt modelling was not used to predict limits to the range of a taxon [55]; ecological descriptors were obtained instead, as suggested by Ruiz et al. [56]. Thus, ecological descriptors were determined for each taxon in terms of monthly, annual and seasonal climatic ranges. Climatic ranges were established once the values for each variable were specified at every site using GIS pixel-value extraction procedures. The extreme values (maximum and minimum) for each variable were determined (in a Microsoft Excel matrix) to establish the climatic ranges and hence the ecological descriptors for teosinte taxa.

## Results and discussion

### Distribution and abundance of teosinte

**Fig 1:**
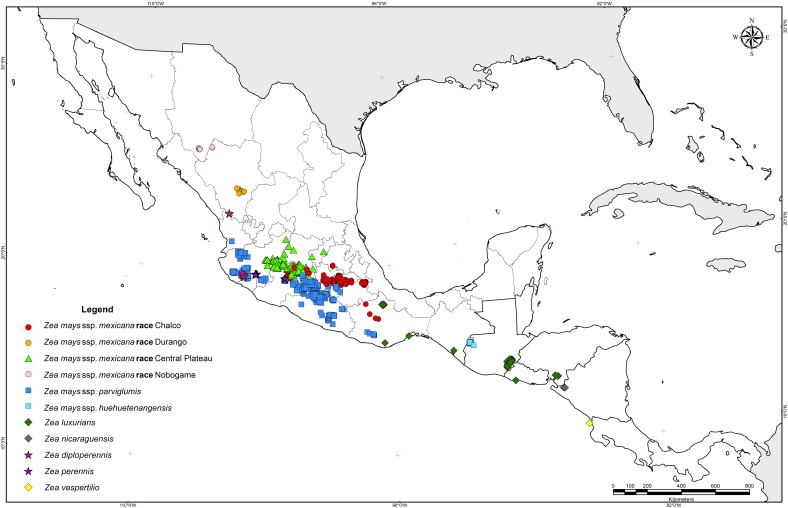
Occurrence records for teosinte species compiled from field observations and collection specimens.

Historical collection data provided the basis to determine the natural distribution of teosinte. Herbarium specimens from the 19^th^ century, documents dating to the 16^th^ century ([6]; Francisco Hernandez in his “Historia de las plantas de Nueva España” written about 1572–1577; Friar Bernardino de Sahagún in his “Historia General de las Cosas de la Nueva España”, written about 1570), and collecting surveys from the 1930´s to year 2010 indicate that the natural geographic distribution of teosinte extends from the southern part of the Western Sierra Madre of the State of Chihuahua and the Guadiana Valley in Durango, to the Pacific coast of Nicaragua and Costa Rica (Table 2; Fig 1) [3, 57, 6, 7, 16, 20]. A total of 1,114 occurrence records for *Zea mays* ssp. *parviglumis*, 772 for *Zea mays* ssp. *mexicana*, 164 of *Zea diploperennis*, 114 for *Zea luxurians*, 97 for *Zea perennis*, 65 for *Zea mays* ssp. *huehuetenangensis*, 24 for *Zea nicaraguensis* and 1 for *Zea vespertilio* were compiled; in addition, no data on taxa were available for 12 records.

**Table 2:** Number of reports for teosinte occurrences in Mexico and Central America.

Taxon	Region	Herbarium	Seed	Document	Archaeology	TOTAL
*Zea mays* ssp. *mexicana*	Chalco	135	231	41	2	**409**
*Zea mays* ssp. *mexicana*	Durango	22	19	2		**43**
*Zea mays* ssp. *mexicana*	Central Plateau	73	190	22		**285**
*Zea mays* ssp. *mexicana*	Nobogame	6	25	4		**35**
*Zea mays* ssp. *parviglumis*	Balsas	178	680	256		**1114**
*Zea mays* ssp. *huehuetenangensis*	Huehuetenango	15	50			**65**
*Zea luxurians*	Guatemala	37	57			**94**
*Zea luxurians*	Oaxaca	3	3	8		**14**
*Zea luxurians*	El Salvador	1				**1**
*Zea luxurians*	Brazil			5		**5**
*Zea nicaraguensis*	Nicaragua	10	14			**24**
*Zea diploperennis*	Jalisco	126	24			**150**
*Zea diploperennis*	Nayarit	1	13			**14**
*Zea perennis*	Jalisco	78	15			**93**
*Zea perennis*	Michoacan		4			**4**
*Zea vespertilio*	Costa Rica	1				**1**
Unclassified		5		2	5	**12**
	**TOTAL**	**691**	**1325**	**340**	**7**	**2363**

Among the 2,363 records, *Zea diploperennis*, *Zea luxurians*, *Zea perennis*, *Zea mays* ssp. *huehuetenangensis*, *Zea vespertilio* and *Zea nicaraguensis* were spatially distinct, representing 19.6% of the total occurrences. The greatest species diversity was observed in western Mexico. The perennial diploids are distributed in very small populations. *Zea diploperennis* grows exclusively in two regions: (i) a small valley in the mountains of the Sierra Madre Occidental, in the northern part of the county of Huajicori, Nayarit at an average altitude of 1400 m, (ii) at the base of rocky north-northeast slopes of Cerro de San Miguel, Manantlan, Las Joyas, Municipality of Cuautitlan, Jalisco (east end of Sierra de Manantlan), at altitudes from 1400 to 2400 m. The perennial tetraploid populations (*Zea perennis*) are restricted to El Fresno, 10 km east of Uruapan, Michoacan at an average altitude of 1380 m; the second area is on the northern slopes of Volcan de Colima in the state of Jalisco at altitudes of 1600–2200 m. *Zea luxurians* is an annual native to southeastern Guatemala, Honduras, El Salvador and southern Mexico at altitudes between sea level and 1100 m. It is also known from a collection made by Liebmann, F.M. in San Agustin, Oaxaca, Mexico in 1842; there are two additional specimens, Soconusco, Chiapas, and San Mateo del Mar, Oaxaca, although no one has reported seed collections from these localities. *Zea luxurians* was introduced to Brazil through public institutions for use as a forage crop by mid 20^th^ century [58].

*Zea nicaraguensis* is a geographically isolated annual teosinte from the coastal plain and estuaries near the Gulf of Fonseca, Nicaragua at elevations of 9 to 75 m; most small populations are restricted to the Department of Chinandega at Rancho Apacunca, Cayanlipe and El Rodeo [21]. *Zea mays* ssp. *huehuetenangensis* is only found in western Guatemala at elevations of 900–1650 m at San Antonio Huista, Jacaltenango, Santa Ana Huista and Nenton. *Zea vespertilio* is a very small population found only in the Murcielago Islands, Santa Elena Peninsula, Guanacaste province of Costa Rica [20].

*Zea mays* ssp. *parviglumis* and *Zea mays* ssp. *mexicana* occupy a diverse geographic range in Mexico; they show some level of geographic overlap within their native range in central and northern Mexico. *Zea mays* ssp. *parviglumis* is found along the western escarpment of the Sierra Madre del Sur, from Nayarit to Oaxaca, Mexico, at elevations between 150 and 1950 m. *Zea mays* ssp. *mexicana* is found in the highlands of central and northern Mexico at altitudes from about 1500 to 2990 m. Race Chalco is found in the Toluca Valley and Chalco-Amecameca, Mexico State and Ciudad Serdán and Puebla in Puebla State. Race Central Plateau occurs in the states of Guanajuato, Michoacan and Jalisco. Race Nobogame is restricted to southern Chihuahua, however, it was also reported for northwestern Durango [59, 60] and Maycoba, Sonora [61]; although for this site, no herbarium or germplasm accession exists. Race Durango is found near the city of Durango and in the county of Nombre de Dios, Durango.

### Dispersion of teosinte as a fodder plant of economic value and as a dangerous weed

**Fig 2:**
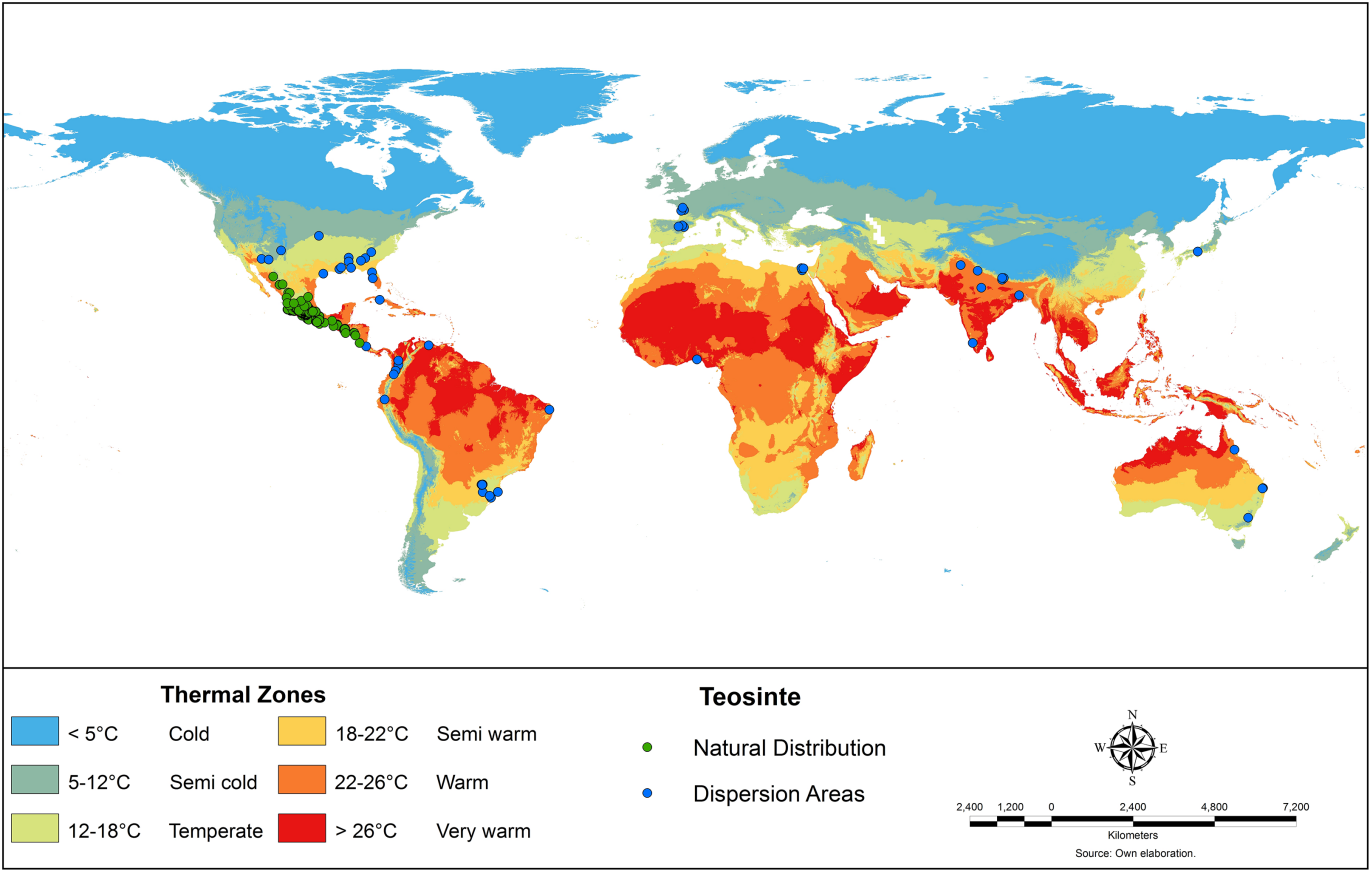
Natural distribution and dispersion areas of teosinte in the world.

Historical occurrence data were also used to show the dispersion of teosinte around the world as fodder plant and as an invasive plant (See Fig 2). A very detailed analysis of teosinte as forage was given by Wilkes [6]. As reported by this author, the French and the English are responsible for the worldwide distribution of teosinte seed during the second half of the 19^th^ century; *Zea luxurians* (*Euchlaena luxurians*) from Guatemala seems to be the original source [6, 62]. Some important quantities of seed were supplied from Egypt and India to the West lndies, Cyprus, South and tropical Africa, Australia, the United States, Guyana (British Guiana) and New Zealand [6, 62]. In India, teosinte is highly valued as silage, it is considered an excellent multi-cut fodder, it has advantages over fodder maize including multiple cutting, high nutritive value, and ease of cultivation. Teosinte is locally known there as “Makchari” and “Makiya”, and several varieties are available, which are planted on about 10,000 ha [63, 64].

Forage crops are very important in Egypt, they contribute about 20% of the total value of field crops. Teosinte is known as “Rayana”, and is considered one of the most promising multicut fodder crops of warm regions. The cultivated area of forage teosinte is increasing; presently, it is grown on about 21,000 ha in Egypt [65, 66]. Green forage is important for milk production in cows and buffaloes in Asia; teosinte is considered a promising summer-forage crop in Nepal, because it is a tall and vigorously growing crop, which can tolerate moderate drought and temporary flooding [67]. In the US, teosinte has been used as forage in several southern states: Louisiana, Mississippi, Georgia, Florida, Texas and Kansas [68, 69, 70]. The earliest presence of wild relatives of maize in South America is well documented for Brazil; however, there is no accurate information about the origin of the current populations. In Santa Catarina State, in South of Brazil, teosinte is commonly called “Guatemalan teosinte”, “Venezuelan grass” and “Imperial grass”, “Teosinto” or “Dente de burro” by farmers. The occurrence of teosinte populations in maize fields in Santa Catarina was recorded in 2011; however, local farmers reported that it has been present since 1949. As in Mexico, most farmers in this region of the country consider teosinte undesirable; however, for others, it represents food safety for many agricultural communities, because the presence and distribution of teosinte is related to its use for grazing, especially by dairy cattle, which is the main economic activity in the region [58].

Several reports in the past 20 years indicate that teosinte has appeared in Spain and France and is spreading widely in maize growing areas as an invasive species [71]. Several field and greenhouse experiments are being developed to find control measures [72, 73]. In several regions of Mexico, teosinte (*Zea mays* ssp. *mexicana*) has become an important weed in maize fields; it has been reported as a problem in the Toluca Valley, in eastern Jalisco (part of the Central Plateau), in the states of Durango, Puebla and Hidalgo, and in several maize fields in the Chalco Valley in the state of Mexico. Some selected references on teosinte as a weed are: Espinosa and Sarukhán [74], Vibrans and Estrada [75], Balbuena et al. [76] and Sánchez-Ken et al. [77].

### Climatic adaptation

Univariate analysis of variance was used initially to select the first set of 45 out 216 climatic variables. Variable selection for clustering and classification (VSCC) was then used to select a final set of 23 variables (Table 3). The inclusion of growing season parameters in the selected variables showed that they are a competitive alternative to the very well-known annual, seasonal and monthly “bioclimatic variables” proposed by Hijmans et al. [39] which are commonly used in describing wild plant species distribution.

**Table 3:** Eigenvectors of 23 climatic variables from principal component analysis of teosinte locations.

Variable	PC1	PC2	PC3
X3 -altitude (m)	-0.2445	-0.0179	-0.0736
X30-annual mean maximum temperature (°C)	0.2487	-0.0508	-0.0914
X44-annual mean minimum temperature (°C)	0.2466	0.0702	-0.0136
X58-annual mean temperature	0.2527	0.0145	-0.0506
X156-annual mean relative humidity (%)	-0.0048	0.4291	-0.1555
X162-mean solar radiation in May	-0.0126	-0.4142	0.2562
X170-annual mean solar radiation	0.0516	-0.4063	0.1580
X175-GS mean maximum temperature (°C)	0.2422	-0.0751	-0.0798
X176-GS maximum temperature (°C)	0.2333	-0.0859	-0.0641
X177-monthly maximum temperature (°C)	0.2389	-0.1066	-0.1033
X178-GS mean minimum temperature (°C)	0.2516	-0.0086	-0.0122
X179-GSn minimum temperature (°C)	0.2451	-0.0204	-0.1057
X180-monthly minimum temperature (°C)	0.2330	0.1146	-0.0040
X181-GS mean temperature (°C)	0.2503	-0.0407	-0.0448
X182-growing cumulative growing-degree days	0.2330	0.1145	0.1662
X187-total growing season precipitation (mm)	0.1376	0.1791	0.7054
X201-growing season length (days)	0.1027	0.3278	0.4910
X208-mean temperature of wettest quarter (°C)	0.2430	-0.0076	-0.0430
X209-mean temperature of driest quarter (°C)	0.2454	0.0527	-0.1103
X210-mean temperature of warmest quarter (°C)	0.2400	-0.0096	-0.1165
X211-mean temperature of coldest quarter (°C)	0.2445	0.0644	-0.0577
X212-Precipitation seasonality (%)	0.1401	-0.2966	0.0350
X218- relative humidity of the driest month (%)	-0.0199	0.4288	-0.1894
Eigenvalues proportions	67.40%	21.70%	4.50%

A principal components analysis (PCA) of the sites using these 23 quantitative climatic variables reveals important patterns of variation among teosinte species in their climatic adaptation. The first three principal components (PCs) accounted for 93.5% of the variation in the ecogeographical data. PC1 explained 67.4% of the variation and is mostly influenced by temperature variables and altitude (Table 3). PC2 explained 21.7% and had strongly positive scores for relative humidity and negative values for solar radiation variables; PC3 explained 4.5% and had strongly positive scores for rainfall and growing season-derived variables (Table 3).

**Fig 3:**
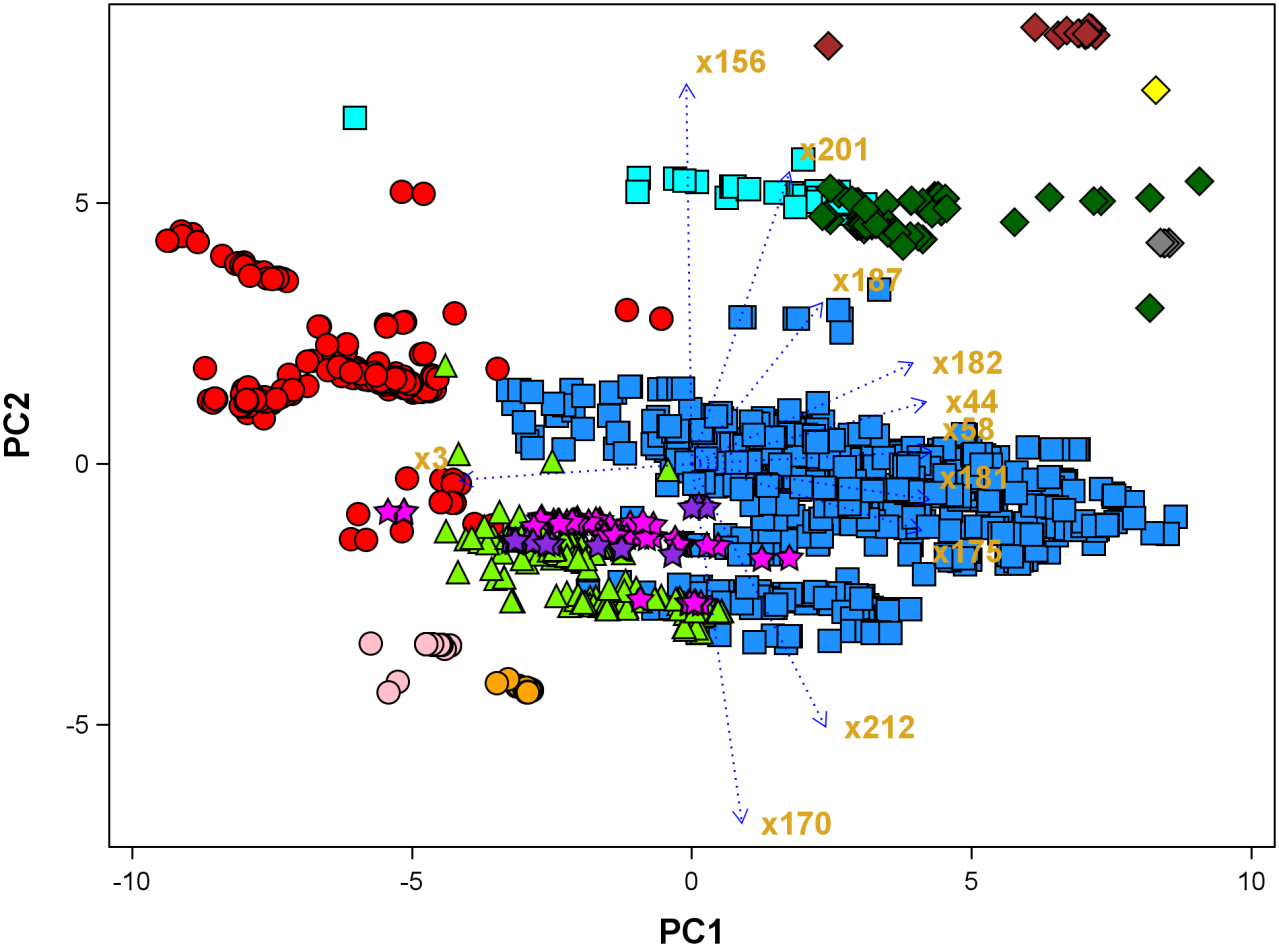
Principal component analysis of collecting sites of teosinte using ecogeographic data (*Zea mays* ssp. *parviglumis* designated with a blue square; *Zea mays* ssp. *mexicana* race Chalco designated with a red circle, race Durango designated with an orange circle, race Central Plateau designated with a lawngreen triangle, race Nobogame designated with a pink circle; *Zea mays* ssp. *huehuetenangensis* designated with a cyan square; *Zea perennis* designated with a purple star; *Zea diploperennis* designated with a magenta star; *Zea luxurians* designated with a darkgreen diamond; *Zea nicaraguensis* designated with a gray diamond; *Zea luxurians* from San Felipe Usila, Oaxaca designated with a brown diamond; *Zea vespertilio* designated with a yellow diamond).

It is important to state that the hierarchical system of classification for *Zea* was mostly based on the morphological features of the tassels, because these have not been under human selection [17, 18]. On the other hand, the races of teosinte are geographical populations spatially isolated by the topography in Mexico and Central America. The ecological conditions of these areas have special characteristics and several physiologically different races have developed, each of which has acquired a limited morphological, ecological, chromosomal, and genetical distinctness [6]. Plotting teosinte occurrences against the first two principal components of climate and geographic data revealed that most sites for *Zea mays* ssp. *parviglumis*, fall in the central part of the biplot graph, suggesting similar adaptations; while race Central Plateau of *Zea mays* ssp. *mexicana* and the perennial teosintes seem to be intermediate among ssp. *parviglumis* and races Durango and Nobogame of ssp. *mexicana* (Fig 3). However, *Zea luxurians* from San Felipe Usila, Oaxaca and Soconusco, Chiapas, *Zea nicaraguensis* and *Zea vespertilio* are very well separated from the main group and displaced in the positive direction of PC1 and PC2, indicating adaptation to a very high rainfall and hot temperatures (highest values of mean temperature, humidity and rainfall, x44, x58, 181, 182, 187, Table 3 and Fig 3). Race Chalco of ssp. *mexicana* is displaced in the negative direction of PC1, indicating an adaptation to moderate rainfall, low temperatures and high altitude (x3 and negative x175, x177, x181). PC2 was dominated by relative humidity (x156), length of the growing season (x201), precipitation seasonality (x212) and solar radiation (x162, x170); this dimension clearly separates *Zea luxurians* from Oaxaca, *Zea vespertilio*, *Zea nicaraguensis*, *Zea luxurians* and *Zea mays* ssp. *huehuetenangensis* on the positive side and races Durango and Nobogame of ssp. *mexicana* on the negative side (Fig 3). These two races are adapted to high altitude, higher solar radiation and low rainfall during shorter growing seasons.

A discriminant analysis was conducted based on the classifications presented in Table 1 and Fig 3. Results of posterior probabilities of membership based on the climatic variables indicated that 95.7% of records were correctly classified into the 14 taxa. Despite the complexity of teosinte diversity, only 4.9% of sites of *Zea mays* ssp. *parviglumis*, 11% of *Zea mays* ssp. *mexicana* (7% of race Chalco, 3% of race Nobogame and 1% of race Central Plateau) and 5.2% of *Zea luxurians* from Guatemala, were misclassified; all remaining races and species were classified without error.

The classification errors observed for *Zea mays* ssp. *parviglumis* and *Zea mays* ssp. *mexicana* race Chalco are due to the occurrence of these races in sites with climatic conditions similar to those associated with other teosinte taxa. For example, several occurrence sites for *Zea mays* ssp. *parviglumis* have different climatic conditions compared to the rest of the Balsas River basin: those in the state of Morelos have climatic conditions more like those of the Chalco area; some from the Sierra de Manantlan have conditions suitable for *Zea diploperennis* and *Zea perennis*, while some locations close to Uruapan have conditions similar to the areas where the tetraploid perennial teosinte is growing in the state of Michoacan.

Similarly, overlap of climatic conditions with other taxa was observed for *Zea mays* ssp. *mexicana* race Chalco. Some populations classified as Chalco race based on morphology were collected around Ciudad Hidalgo in the State of Michoacan, where the climate is more like the Central Plateau. These results agree with the concept of “geographic races” of Wilkes [6, 8] and the classification criteria used in this study (Fig 3 and Table 1). Based on this classification, the discriminant analysis indicates that the report of teosinte in Yecora, Sonora should be classified as *Zea mays* ssp. *mexicana* race Nobogame, while that of Cerro Prieto in Durango should be classified as *Zea mays* ssp. *mexicana* race Durango.

Table 4 shows the ecological descriptors for 14 teosinte taxa in terms of environmental ranges. Teosinte grows in a wide variety of ecological conditions, with differences in taxa distributions mainly influenced by growing season climate factors and altitude. Extremes in growing season length (GSL) are represented by race Durango of ssp. *mexicana* (83 days) and *Z*. *luxurians* from San Felipe Usila, Oaxaca (303 days); the range of altitude in teosinte distribution is from almost sea level (Z. *vespertilio*) to 2990 m (race Chalco of ssp. *mexicana*).

**Table 4:** Range of ecological descriptors (environmental intervals) for 14 teosinte taxa.

Taxon	Region		Growing season					Annual	
		Altitude	Length	Mean temperature	Rainfall	Minimum temperature	Maximum temperature	Mean temperature	Rainfall
		(m)	(days)	(°C)	(mm)	(°C)	(°C)	(°C)	(mm)
*Zea mays* ssp. *mexicana*	Chalco	1700–2990	119–303	13.0–21.3	312–1148	4.5–14.5	19.1–29.0	12.3–20.5	451–1321
*Zea mays* ssp. *mexicana*	Durango	1860–1950	83–93	19.8–20.4	305–339	12.2–12.7	27.3–28.1	16.7–17.4	468–512
*Zea mays* ssp. *mexicana*	Central Plateau	1500–2208	114–153	17.4–22.3	305–860	8.3–14.8	26.2–33.3	16.2–20.4	458–988
*Zea mays* ssp. *mexicana*	Nobogame	1850–2020	97–123	17.7–22.8	443–800	6.1–13.7	26–31.4	13.9–17.2	670–1088
*Zea mays* ssp. *parviglumis*	Balsas	143–1960	130–185	17.8–28.4	557–1475	10.4–21.3	24.9–37.8	17.1–28.3	698–1521
*Zea mays* ssp. *huehuetenangensis*	Huehuetenango	860–2500	194–243	15.5–23.9	1115–1431	6.9–15.0	23.3–32.7	15.3–23.2	1193–1600
*Zea luxurians*	Guatemala	4–1200	148–216	23.0–28.4	824–2744	16.6–22.1	30.3–35.4	22.5–28.2	886–2864
*Zea luxurians*	Oaxaca	40–250	292–303	21.0–25.4	3503–3669	13.2–16.3	27.9–33.0	21.2–25.6	3629–3805
*Zea nicaraguensis*	Niacaragua	9–15	205–207	27.7–27.8	1535–1622	20.6–20.9	35.5–35.5	27.7–27.8	1576–1667
*Zea diploperennis*	Jalisco	1350–2300	147–160	16.4–24.1	905–1229	10.0–16.4	23.9–32.6	15.6–22.7	1024–1364
*Zea diploperennis*	Nayarit	1390–1410	148–149	20.5–21.6	1270–1289	12.7–13.7	29.0–30.2	18.6–19.6	1412–1433
*Zea perennis*	Jalisco	1500–2174	146–148	18.6–21.4	740–771	11.0–13.3	26.6–29.6	17.4–19.9	846–891
*Zea perennis*	Michoacán	1380–1385	158–159	21.2–21.5	1049–1087	12.7–13.0	29.4–29.7	20.2–20.4	1186–1224
*Zea vespertilio*	Costa Rica	3	219	27.3	1632	22.6	33.1	27.8	1620

**Fig 4:**
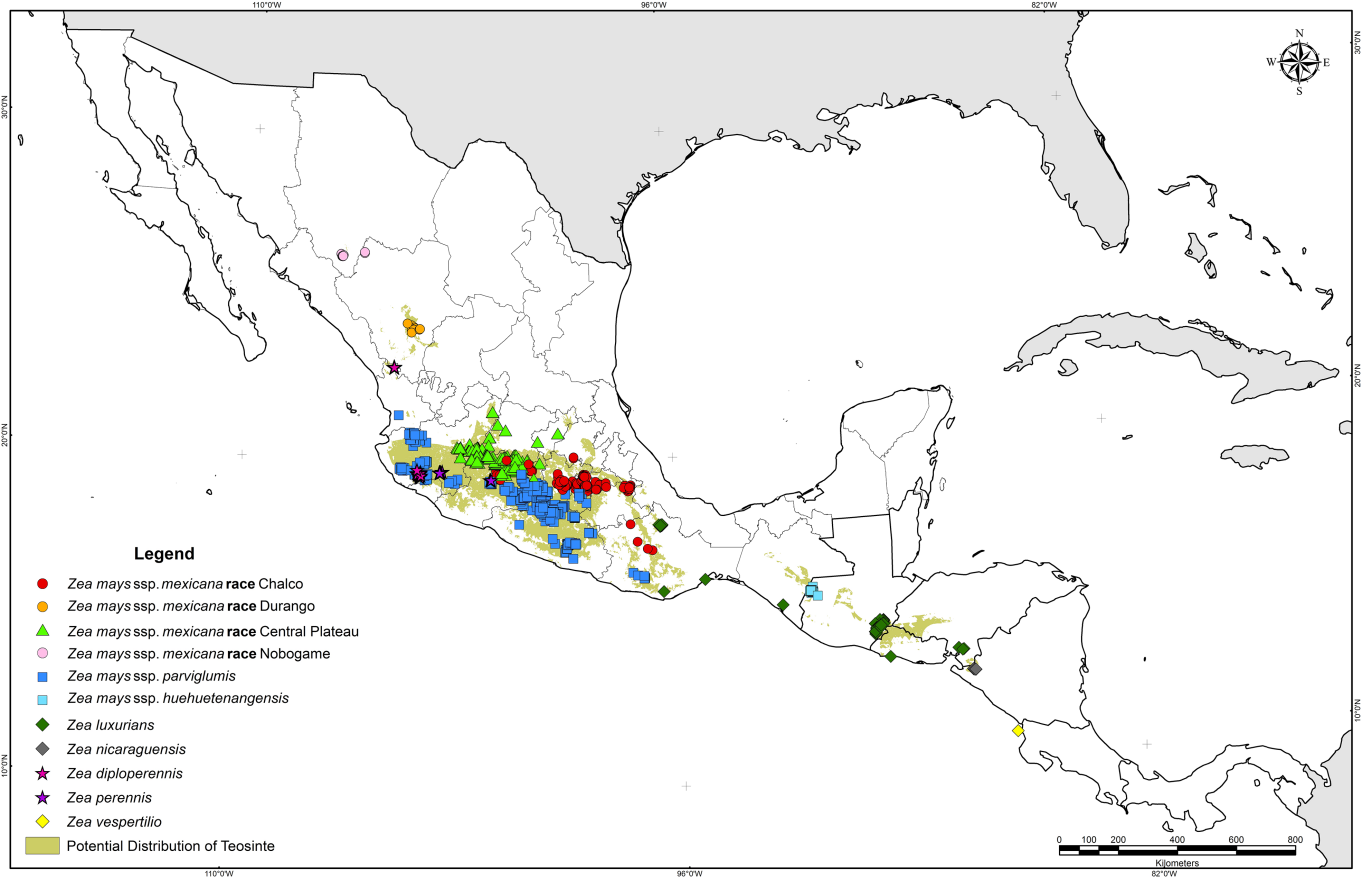
Actual and potential distribution of teosinte.

Teosinte taxa can grow with mean minimum temperatures as low as 4.5°C (*Zea mays* ssp. *mexicana* race Chalco) and mean maximum temperatures as high as 37.8°C (*Zea mays* ssp. *parviglumis*). In addition, some teosinte taxa vary in their adaptation to rainfall from regions with 305 mm accumulated during the growing season (*Zea mays* ssp. *mexicana* races Durango and Central Plateau) to regions receiving 3669 mm (*Z*. *luxurians* from San Felipe Usila, Oaxaca). These wide intervals in climatic variables explain the great adaptability of teosinte and its great capabilities to disperse to new agroecological areas (Fig 4).

The adaptation of teosinte to environmental conditions that would be considered abiotic stresses in an agronomic context, suggests that these populations could harbor unique and favorable genes that could be transferred to new maize varieties to improve their adaptation to stressful environments that may become more common due to climate change. Ecogeographic analysis of the local and regional distribution of teosinte taxa in Mexico and Central America highlights the distinctiveness of several teosinte taxa with respect to environmental adaptation.

Comparing these extreme ecological descriptors to those from maize in Mexico [56] suggests that teosinte has a higher maximum altitude (2990 m) than maize (2900 m, landraces Chalco and Cónico), a lower growing season rainfall threshold than maize (304 mm for teosinte, 400 mm for maize landraces Bolita, Cacahuacintle, Chalqueño, Cónico, Cónico Norteño, Elotes Cónicos, Ratón and Tuxpeño Norteño); and a higher rainfall maximum (3669 mm for teosinte; 3555 mm for maize landrace Comiteco). Maize has a higher threshold for mean temperature during the growing season (29.1°C for landrace Tuxpeño) than teosinte (28.4°C, Balsas). These values suggest maize and teosinte have similarly wide ranges of adaptation, although since maize has a much wider geographic range than teosinte, maize can certainly grow in colder and shorter growing seasons [78, 79]; furthermore, some maize varieties are adapted to long day length growing seasons.

### Potential distribution for teosinte

Distribution models obtained for all teosinte taxa showed an AUC value superior to 0.95 (Table 5), indicating a good discrimination of suitable versus unsuitable areas for teosinte [47, 80]. Moreover, all models were significantly better than random in binomial tests of omission (P< 0.01). The optimal thresholding method to generate binary maps varied among teosinte taxa studied, but in 5 of 10 cases the tenth percentile training presence 10PTP was the best option (Table 5).

**Table 5:** Summary statistics of the models for teosinte taxa. AUC for: (a) training data and (b) testing data; method selected for thresholding; Logistic threshold to obtain the binary map (suitable and unsuitable areas for teosinte distribution), and omission rate of the models.

Taxon	Area Under the Curve		Method of thresholding	Logistic threshold	Omission rate
	Training	Testing			
*Zea mays* ssp. *parviglumis* (Balsas)	0.982	0.976	ETSS	0.144	0.011
*Zea mays* ssp. *mexicana* (Chalco)	0.993	0.992	FC10	0.275	0.055
*Zea mays* ssp. *mexicana* (Central Plateau)	0.993	0.992	10PTP	0.271	0.072
*Zea mays* ssp. *mexicana* (Durango)	0.998	0.998	10PTP	0.609	0.000
*Zea mays* ssp. *mexicana* (Nobogame)	0.998	0.998	10PTP	0.332	0.025
*Zea mays* ssp. *huehuetenangensis*	0.998	0.998	10PTP	0.416	0.047
*Zea luxurians*	0.996	0.990	EETOD	0.221	0.058
*Zea nicaraguensis*	0.998	0.998	MTP	0.539	0.014
*Zea diploperennis*	0.998	0.997	MTP	0.240	0.019
*Zea perennis*	0.997	0.996	10PTP	0.525	0.086

ETSS = Equal training sensitivity and specificity; FC10 = Fixed cumulative value 10; 10PTP = 10 percentile training presence; EETOD = Equal entropy of thresholded and original distributions; MTP = Minimum training presence.

Binary maps revealed that at large scale the simulated current distribution matched actual distribution ranges of teosinte (Fig 4). However, at a small scale many areas within the potential range have no reported teosinte, which could have important implications for searching for and finding new teosinte populations in the near future.

Jackknife analysis reported that the variables with highest weight in the models of potential distribution were GS (growing season length) for *Zea mays* ssp. *parviglumis*, *Zea mays* ssp. *mexicana* race Durango, *Zea mays* ssp. *huehuetenangensis* and the complex of teosinte (all taxa combined); GDD (growing degree-days) accumulated in GS for *Zea diploperennis*, *Zea perennis* and *Zea luxurians*; mean annual relative humidity for *Zea mays* ssp. *mexicana* race Central Plateau; Bio01 (annual mean temperature) for *Zea nicaraguensis*; mean annual minimum temperature for *Zea mays* ssp. *mexicana* race Nobogame, and Bio08 (mean temperature of wettest quarter) for *Zea mays* ssp. *mexicana* race Chalco.

**Fig 5:**
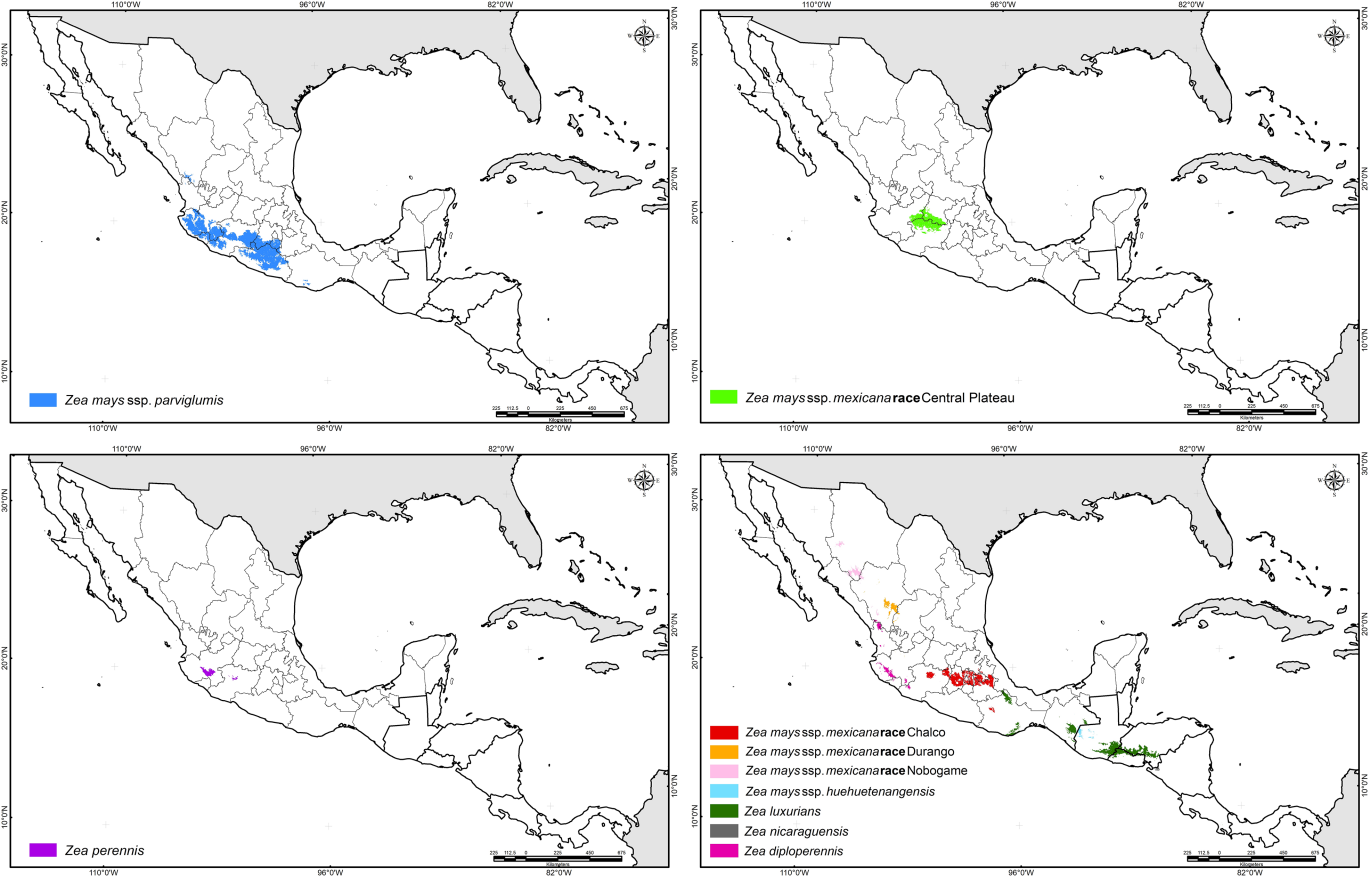
Potential distribution of teosinte taxa.

Potential areas for *Zea mays* ssp. *mexicana* races Chalco, Nobogame and Durango, *Zea mays* ssp. *huehuetenangensis*, *Zea luxurians*, *Zea diploperennis* and *Zea nicaraguensis* are geographically separated, whereas the distribution of *Zea mays* ssp. *parviglumis* overlaps with *Zea mays* ssp. *mexicana* race Central Plateau, *Zea perennis*, and *Zea diploperennis* (Fig 5). Suitable areas for *Zea luxurians* are relatively extended and encompass a territory from Oaxaca, Mexico to the border between Honduras and Nicaragua, including areas in Oaxaca, Chiapas, Guatemala, Honduras and El Salvador (Fig 5). The cases of *Zea nicaraguensi*s and *Zea mays* ssp. *huehuetenangensis* constitute the most constrained potential areas, mainly because of their very specific geographical distributions (Fig 5); consequently, only environmental variable values within quite restricted ranges constitute suitable areas for these taxa [47].

The suitable areas for *Zea mays* ssp. *mexicana* races Nobogame and Durango are very similar to their current distribution and very small surrounding areas. Race Nobogame reached the farthest north, to near latitude 29° (Fig 5). Both Nobogame and Durango races constitute subtropical teosintes because of their distribution beyond the Tropic of Cancer (23° 27’). *Zea diploperennis* is restricted in its distribution to the western portion of Mexico, primarily in the states of Jalisco and Nayarit, matching its actual distribution; however, an interesting potential area is located at the border between south-eastern Jalisco and south-western Michoacán where searches for new populations could be prioritized. *Zea perennis* has been collected in only a few sites in Jalisco and Michoacán, and its potential distribution encompasses an area just surrounding the known sites (Fig 5).

### In-situ and ex-situ conservation

**Fig 6:**
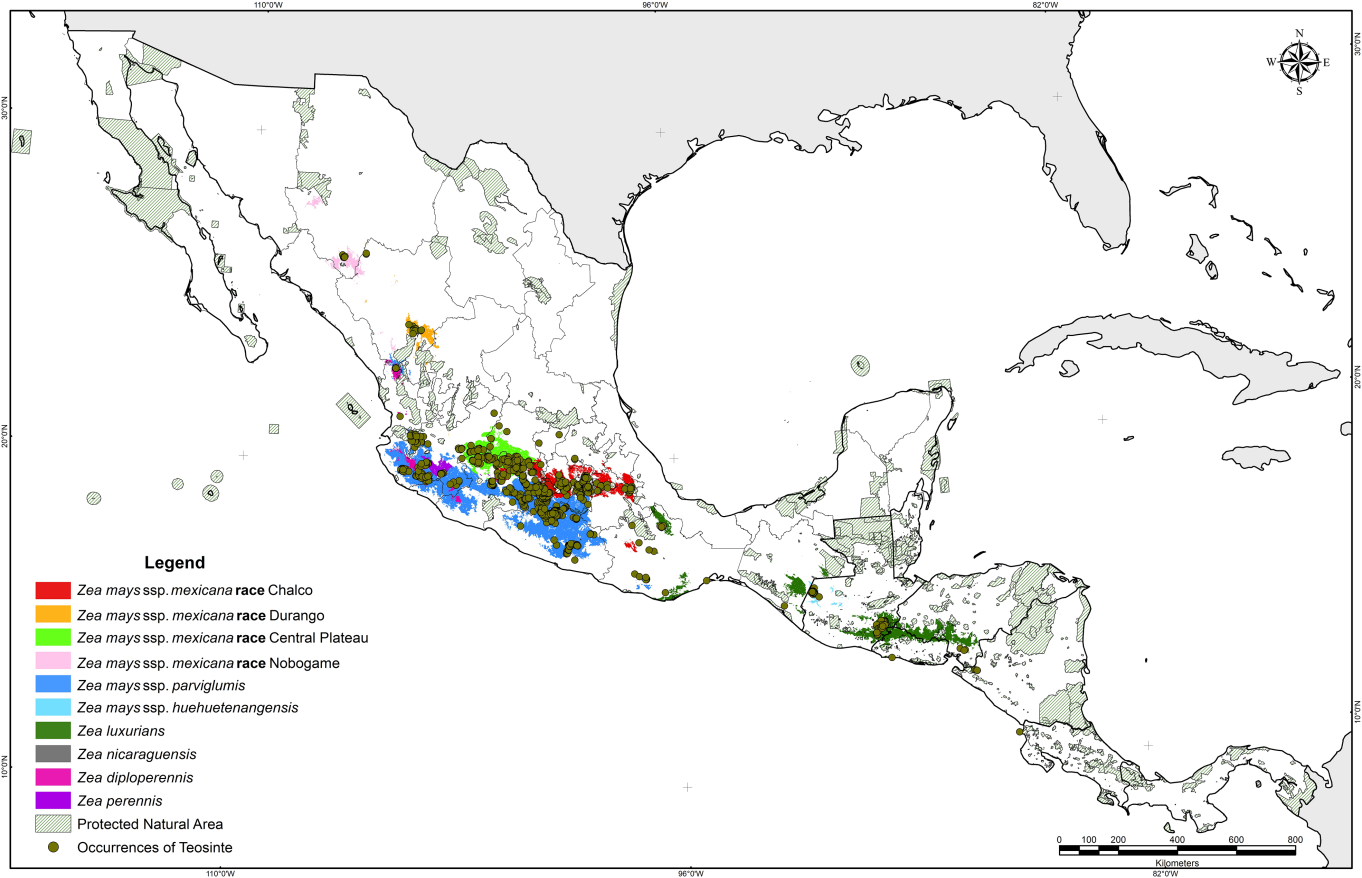
Actual and potential distribution of teosinte and geographical location of PNAs.

When comparing teosinte distribution areas (actual and potential) against the map for Protected Natural Areas (PNA) in Mexico and Central America, only 11.2% of teosinte populations are found in PNAs (Fig 6); moreover, only 1.0, 7.4 and 7.6% of Chalco, Guatemala and Balsas populations are found in PNAs. There are only two PNAs specifically created to conserve teosinte species: The Sierra de Manantlan Biosphere Reserve established in Jalisco, Mexico in 1987 which preserves *Zea diploperennis* and The Apacunca Genetic Reserve in Nicaragua created in 1996 to protect *Zea nicaraguensis*. Of the areas representing current distributions of *Zea nicaraguensis* and *Zea diploperennis*, 62.5 and 98.7% are in PNAs, respectively. In contrast only 0.1% (Central Plateau) to 9.4% (*Zea perennis*) of other teosinte populations are in PNAs (Fig 6).

The most important *ex situ* teosinte collections are held in the germplasm banks of INIFAP, CIMMYT, NPGS and Universidad de Guadalajara. Of these, only NPGS, CIMMYT and INIFAP have long-term storage facilities. The NPGS-GRIN database (January 2017) reports 895 historical accessions, of which 363 are not available, 453 are inactive, and only 80 have seed available for distribution. CIMMYT houses about 300 accessions, INIFAP about 450 and University of Guadalajara stores 515 accessions. Combined, all teosinte collections represent about 3% of global *Zea* accessions. Other Mexican and Central American institutions, including Universidad Autónoma Chapingo and Colegio de Postgraduados from Mexico, Instituto de Ciencia y Tecnología Agrícolas de Guatemala (ICTA) and Instituto Nicaragüense de Tecnología Agropecuaria (INTA), preserve teosinte seed in different quantities and under varied conditions. It is important to stress that most populations have been sampled in small seed quantities, mostly for genetic and morphological studies; thus, availability of seed samples is very limited and uncertain for most institutions. Only NPGS has online passport data; however, for all institutions it is very difficult to access data on seed availability, this hinders the development of global plans for seed regeneration and seed exchange among germplasm banks.

A high priority for conservation of teosinte is to identify those populations reported in documents or as herbarium specimens, but missing from seed collections in national or international germplasm banks. Two studies have reported analysis of the extent of representation of the wild relatives of several crops, including maize, in gene banks [81, 82]. Some problems reported in these studies are: (i) most *Zea perennis* occurrences were misclassified and do not correspond to current populations, (ii) some herbarium specimens of *Zea diploperennis* come from cultivated plants in sites beyond the natural range, and (iii) that whereas for many crops, experts assessed input data and results, lists of experts or institutions conserving teosinte are absent for *Zea*.

Based upon the gap analysis process described by Ramírez-Villegas et al. [83], in this work we estimated the populations and taxa under-represented or absent from germplasm collections using several representativeness scores (Table 6): sampling representativeness score (SRC), geographic coverage (GCS), rarity of each taxa (RTS), and inventory data, when available, were considered. In addition, data on *in situ* monitoring conducted by CIMMYT, INIFAP and Universidad de Guadalajara to evaluate changes in teosinte populations in their natural habitats for the last 40 years [84, 16] provided the information for the experts score (ExS) included in Table 6.

**Table 6:** Assessment of priority for collecting for conservation in gene banks.

Taxon	TOT^1^	BA^2^	POT^3^	SGC^4^	GCS^5^	SRC^6^	RTS^7^	ExS^8^	AVS^9^
*Zea mays* ssp. *mexicana* (Chalco)	409	231	25964	7257	2.8	5.6	2.7	5	4.0
*Zea mays* ssp. *mexicana* (Durango)	43	19	4479	597	1.3	4.4	0	1	**1.7**
*Zea mays* ssp. *mexicana* (C. Plateau)	285	190	21954	5969	2.7	6.7	0	5	3.6
*Zea mays* ssp. *mexicana* (Nobogame)	35	25	5127	785	1.5	7.1	0	1	**2.4**
*Zea mays* ssp. *parviglumis*	1114	680	89789	21363	2.4	6.1	0.7	5	3.5
*Zea mays* ssp. *huehuetenangensis*	65	50	1559	1571	10.1	7.7	0	1	4.7
*Zea luxurians*	94	60	28773	1885	0.7	6.4	1.7	1	**2.4**
*Zea nicaraguensis*	24	14	524	440	8.4	5.8	10	1	6.3
*Zea diploperennis*	150	37	5088	1162	2.3	2.5	0	1	**1.4**
*Zea perennis*	93	19	3732	597	1.6	2	0	1	**1.1**
*Zea vespertilio*	1	0	1	0	0	0	10	1	**2.8**

^1^TOT = Total records,

^2^BA = Bank accessions,

^3^POT = Potential area km^2^,

^4^SGC = Seed geographic coverage (area of 10 km radius per seed accession);

^5^GCS = Geographic coverage score (seed geographic coverage/potential area)*10,

^6^SRC = Sampling representativeness score (number of seed accessions/all taxon records)*10,

^7^RTS = Rarity taxa score (records in rare environments(5 and 95 percentiles)/total records of taxon)*10,

^8^ExS = Experts score (where 1 corresponds to a very high priority, and 10 corresponds to the lowest priority),

^9^AVS = Average score.

The general assessment presented in Table 6 suggests that six out of the 11 taxa with lowest average scores should be considered as high priority for collecting; the remainder as medium priority. It is important to notice that because of limited collecting efforts, the sampling representativeness score is higher than expected for ssp. *huehuetenangensis* and *Zea nicaraguensis*. Although it is difficult to estimate danger of extinction and relative threat [10, 11, 16], the largest teosinte populations have become fragmented and significantly diminished, some populations are endangered and cannot be expected to persist much longer. For example, no wild populations remain for *in situ* preservation in Guatemala and several are already extinct. Most of the perennial teosintes and many of the tropical *Zea mays* ssp. *parviglumis* populations are vulnerable because of cattle farming, the establishment of pastures, the introduction of mechanical tilling and fruit and avocado orchards in the natural distribution of the perennial populations. Although the status of weedy teosinte populations (*Zea mays* ssp. *mexicana* races Chalco and Central Plateau) has been considered ‘indeterminate’ and ‘stable’ for *Zea mays* ssp. *parviglumis* [11], urbanization, introduction of modern maize hybrids, and the use of herbicides among other human activities are affecting the stability of the populations.

Because of these threats, permanent monitoring programs and *in-situ* conservation projects with participation of local farmer communities are critically needed. Among the 2363 records considered in this study, about 400 are not represented in the most important existing collections or have limited seed quality and quantity. Among these, 250 can be considered unique populations or “fragments”. Based on sampling representativeness scores, geographic coverage and monitoring information, collection and *ex situ* conservation activities are urgently and immediately needed in Guatemala, Costa Rica, Durango, Chihuahua and all sites of perennial teosintes in Mexico. In the long term, sampling for long-term conservation and *in situ* monitoring and protection will be required for most populations.

## Conclusions

A good first step to determining potential breeding value and priorities for conservation of crop wild relatives would be the creation of a reliable database including the historical occurrence of all taxa. In this work on teosinte of Mexico and Central America, the detailed review of occurrence records, standardization of the taxonomy and the assembling of a climatological database facilitated a robust ecogeographic study and high precision modelling of the current and potential distribution of teosinte taxa. There are 14 teosinte taxa native to Mexico and Central America, adapted to a very broad range of environmental conditions. The adaptation of teosinte to environmental conditions that would be considered abiotic stresses in an agronomic context suggests that these populations could harbor unique and favorable genes that could be transferred to new maize varieties to improve their adaptation to stressful environments that may become more common due to climate change. Ecogeographic analysis of the local and regional distribution of teosinte taxa in Mexico and Central America highlights the distinctiveness of several teosinte taxa with respect to environmental adaptation. This information will guide researchers to identify the most appropriate places for the regeneration of accessions, to design *in situ* conservation programs, and identify new sources of germplasm to breed maize to withstand a wide array of biotic and abiotic stresses.

Potential geographical distributions, even those developed from limited numbers of occurrence records, may be valuable in designing field surveys to accelerate the discovery of unknown populations and species of teosinte. However, models developed using small sample sizes should be interpreted as identifying regions that have similar environmental conditions to where the species is known to occur, and not as predicting actual limits to the range of a species. We also observed that growing season parameters were more important than monthly or annual climate summary statistics for explaining current distributions and predicting potential distributions of teosinte. Thus, the inclusion of growing season parameters in the geographical information system developed in this study was critical. This information system is a valuable source of agroclimatic information to model species distributions in Mexico and Central America.

## Supporting information

S1 Fig Weather stations considered in the construction of the information system. (TIF)

S2 Fig Agroclimatic regions for the study area. (TIF)

## References

[1] Sarukhán J, Halffter G, Koleff P, González R, Carabias J, March I et al. Capital natural de Mexico. Síntesis: conocimiento actual, evaluación y perspectivas de sustentabilidad. Comisión Nacional para el Conocimiento y Uso de la Biodiversidad, Mexico. 2009.

[2] Food and Agriculture Organization of the United Nations (FAO). The Second Report on the State of the World’s Plant Genetic Resources for Food and Agriculture. Rome. 2010; 370 p.

[3] Collins GN. Teosinte in Mexico. Journal of Heredity. 1921; 12: 339–350.

[4] Kempton JH and Popenoe W. Teosinte in Guatemala: Report of an expedition to Guatemala, El Salvador, and Chiapas, Mexico. Carnegie Institution of Washington. Contributions to American Archaeology. 1937; 4: 201–219.

[5] Mangelsdorf PC. Corn: Its origin, evolution and improvement. Belknap Press. Harvard University Press Cambridge, Mass 1974; 262 p.

[6] Wilkes HG. Teosinte: the closest relative of maize. Cambridge, Massachusetts: The Bussey Institute, Harvard University 1967; 159p.

[7] Kato YTA. Cytological studies of maize (*Zea mays* L.) and teosinte (*Zea mexicana* Schrader Kuntze) in relation to their origin and evolution. Mass. Agr. Exper. Stat. Bull. 635 1976; 186p.

[8] Wilkes HG. Hybridization of maize and teosinte in Mexico and Guatemala and the improvement of maize. Econ Bot. 1977; 31: 254–293.

[9] Wilkes HG. Teosinte: the closest relative of maize revisited. Maydica. 1985; 30(2): 209–223.

[10] Wilkes HG. Teosinte in Mexico: Personal Retrospective and Assessment In: Serratos J.A., Willcox M.C. Castillo y F. (eds.). Gene Flow Among Maize Landraces, Improved Maize Varieties, and Teosinte: Implications for Transgenic Maize. Mexico, D.F. CIMMYT 1997; pp.10–17.

[11] Wilkes HG. Corn, Strange and Marvelous: But Is a Definitive Origin Known? In: CW Smith (Ed) Corn: Origin, History, Technology, and Production. John Wiley & Sons 2004; pp. 3–63.

[12] Sánchez GJJ and Ordaz SL. Systematic and Ecogeographic Studies on Crop Genepools: 2. El teocintle en Mexico. Distribución y situación actual de las poblaciones. IBPGR, Rome. 1987; 50p.

[13] Sánchez GJJ, and Ruiz CJA. Teosinte Distribution in Mexico In: Serratos J.A., Willcox M.C. Castillo y F. (eds.). Gene Flow Among Maize Landraces, Improved Maize Varieties, and Teosinte: Implications for Transgenic Maize. Mexico, D.F. CIMMYT 1997; pp.18–35.

[14] Sánchez GJJ, Kato YTA, Aguilar SM, Hernández CJM, López RA y Ruiz CJA. Distribución y caracterización del teocintle. Libro Técnico Núm. 2. Centro de Investigación Regional del Pacífico Centro, Instituto Nacional de Investigaciones Forestales, Agrícolas y Pecuarias. 1998; 150p.

[15] Ruiz CJA, Sánchez GJJ and Aguilar SM. Potential distribution of teosinte in Mexico: A GIS approach. Maydica. 2001; 46: 105–110.

[16] Sánchez GJJ, De la Cruz LL, Vidal MVA, Ron PJ, Taba S, Santacruz-Ruvalcaba F et al Three new teosintes (Zea spp., Poaceae) from Mexico. Amer. J. Bot. 2011; 98 (9): 1537–1548. 21875968 10.3732/ajb.1100193

[17] Iltis HH and Doebley JF. Taxonomy of *Zea* (Gramineae).II. Subspecific categories in the *Zea mays* complex and a generic synopsis. Amer. J. Bot. 1980; 67: 994–1004.

[18] Doebley JF and Iltis HH. Taxonomy of Zea (Gramineae) I. A subgeneric classification with key to taxa. Amer. J. Bot. 1980; 67: 982–993.

[19] Doebley JF. Molecular evidence and the evolution of maize. Econ Bot. 1990; 44: 6–27.

[20] Gómez-Laurito J. A new species of Zea (Poaceae) from the Murciélago Islands, Santa Elena Peninsula, Guanacaste, Costa Rica. BRENESIA. 2013; 80: 36–39.

[21] Iltis HH and Benz BF. *Zea nicaraguensis* (Poaceae), a new teosinte from Pacific coastal Nicaragua. Novon. 2000; 10: 382–390.

[22] Matsuoka Y, Vigouroux Y, Goodman MM, Sanchez GJJ, Buckler E and Doebley JA single domestication for maize shown by multilocus microsatellite genotyping. Proceedings of the National Academy of Sciences. 2002; 99: 6080–6084. 10.1073/pnas.052125199 PMC122905 11983901

[23] Doebley JF. The genetics of maize evolution. Annual Review of Genetics. 2004; 38: 37–59. doi: 10.1146/annurev.genet.38.072902.092425 15568971 10.1146/annurev.genet.38.072902.092425

[24] Warburton ML, Wilkes G, Taba S, Charcosset A, Mir C, Dumas F et al Gene flow among different teosinte taxa and into the domesticated maize gene pool. Genetic Resources and Crop Evolution. 2011; 58: 1243–1261.

[25] Wilkes HG. Mexico and Central America as a Center for the origin of agriculture and the evolution of maize. Crop Improv. 1979; 6: 1–18.

[26] Hufford MB, Lubinksy P, Pyhajarvi T, Devengenzo MT, Ellstrand NC and Ross-Ibarra J. The Genomic Signature of Crop-Wild Introgression in Maize. PLoS Genet. 2013; 9(5): e1003477 doi: 10.1371/journal.pgen.1003477 23671421 10.1371/journal.pgen.1003477 PMC3649989

[27] Dempewolf H, Eastwood RJ, Guarino L, Khoury CK, Müller JV and Toll J. Adapting Agriculture to Climate Change: A Global Initiative to Collect, Conserve, and Use Crop Wild Relatives. Agroecology and Sustainable Food Systems. 2014; 38:4, 369–377.

[28] Varshney RK, Terauchi R and McCouch S. Harvesting the Promising Fruits of Genomics: Applying Genome Sequencing Technologies to Crop Breeding. Plos Biology. 2014; 12(6): e1001883 doi: 10.1371/journal.pbio.1001883 24914810 10.1371/journal.pbio.1001883 PMC4051599

[29] Mano Y and Omori F. Flooding tolerance in maize (*Zea mays* subsp. *mays*) F1 hybrids containing a QTL introgressed from teosinte (*Zea nicaraguensis*). Euphytica. 2015; 205:255–267.

[30] Nault LR. Origins of leafhopper vectors of maize pathogens in Mesoamerica. In DT Gordon, JK Knoke, LR Nault and RM Ritter [Eds]. Proceedings International Maize Virus Disease Colloquium and Workshop, 2–6 August 1982, pp. 75–82. The Ohio State University, Ohio Agricultural Research and Development Center, Wooster. 1983. USA.

[31] Rich PJ and Ejeta G. Towards effective resistance to Striga in African maize. Plant Signal Behav. 2008; 3(9): 618–621. 19513251 10.4161/psb.3.9.5750 PMC2634541

[32] Lennon JR, Krakowsky M, Goodman M, Flint-Garcia S and Balint-Kurti PJ. Identification of Alleles Conferring Resistance to Gray Leaf Spot in Maize Derived from its Wild Progenitor Species Teosinte. Crop Science. 2016; 56 1: 209–218.

[33] Endresen DTF, Street K, Mackay M, Bari A, Pauw ED. Predictive Association between Biotic Stress Traits and Eco-Geographic Data for Wheat and Barley Landraces. Crop Science. 2016 51:2036–2055.

[34] Eastman JR. Idrisi Selva Manual. Idrisi Project, Clark University. Massachusetts, USA. 2012; 322 p.

[35] Zhang X and Yang F. RClimDex (1.0). User manual. Climate Research Branch Environment Canada. Downsview, Ontario, Canada. 2004; 23 p.

[36] Stöckle CO, Campbell GS and Nelson R. ClimGen manual. Biological Systems Engineering Department, Washington State University, Pullman, WA. 1999; 28 p.

[37] Hutchinson MF. Anusplin Version 4.3: User guide. The Australian National University-Centre for Resource and Environmental Studies, Canberra. 2004; 54 p.

[38] Food and Agriculture Organization of the United Nations (FAO). Agroecological zones project. World Soil Resources. Report Num. 48, Vol. 1: Africa. Geneva, Switzerland. 1978; 158 p.

[39] Hijmans RJ, Cameron SE, Parra JL, Jones PG and Jarvis A. Very high resolution interpolated climate surfaces for global land areas. International Journal of Climatology. 2005; 25: 1965–1978.

[40] SAS Institute Inc. Statistical Analysis System, University Edition. SAS Institute Inc. SAS/IML^®^ 14.1 User’s Guide. 2015. Cary, NC.

[41] Andrews JL and McNicholas PD. Variable Selection for Clustering and Classification. Journal of Classification. 2014; 31:136–153.

[42] Rohlf FJ. NTSYS-pc. Numerical taxonomy and multivariate analysis system, Version 2.1. Exeter Software. 2000; New York.

[43] Phillips SJ and Elith J. Logistic methods for resource selection functions and presence‐only species distribution models. Proceedings of the Twenty‐Fifth AAAI Conference on Artificial Intelligence. 2011; pp. 1384‐1389. San Francisco, USA.

[44] Elith J, Phillips SJ, Hastie T, Dudík M, Chee YE and Yates CJ. A statistical explanation of MaxEnt for ecologists. Diversity and Distributions. 2011; 17: 43‐57.

[45] Fourcade Y, Engler JO, Rödder D and Secondi J. Mapping species distributions with MaxEnt using a geographically biased sample of presence data: A performance assessment of methods for correcting sampling bias. Plos One. 2014; 9(5): e97122 doi: 10.1371/journal.pone.0097122 24818607 10.1371/journal.pone.0097122 PMC4018261

[46] Wisz MS, Hijmans RJ, Li J, Peterson AT, Graham CH and Guisan A. Effects of sample size on the performance of species distribution models. Divers Distrib. 2008; 14: 763–773.

[47] Phillips SJ, Anderson RP and Schapire RE. Maximum entropy modeling of species geographic distributions. Ecological Modelling. 2006; 190(3–4): 231–259.

[48] Avila CA, Villavicencio GR and Ruiz CJA. Distribución potencial de Pinus herrerae Martínez en el Occidente del estado de Jalisco. Rev. Mex. Cien. For. 2014; 5(24): 92–108.

[49] Merow C, Smith MJ and Silander JA Jr. A practical guide to MaxEnt for modelling species’ distributions: what it does, and why inputs and settings matter. Ecography. 2013; 36: 1058–1069.

[50] Hanley JA and McNeil BJ. The meaning and use of the area under a receiver operating characteristic (ROC) curve. Radiology. 1982; 143(1): 29–36. 7063747 10.1148/radiology.143.1.7063747

[51] Soberón J and Peterson T. Ecological niche shifts and environmental space anisotropy: a cautionary note. Revista Mexicana de Biodiversidad. 2011; 82:1348–1355.

[52] Pawar S, Koo MS, Kelley C, Ahmed MF, Chaudhuri S and Sarkar S. Conservation assessment and prioritization of areas in Northeast India: Priorities for amphibians and reptiles. Biol. Conserv. 2007; 136: 346–361.

[53] Donegan TM and Avendaño JE. A new subspecies of mountain tanager in the Anisognathus lacrymosus complex from the Yariguíes Mountains in Colombia. Bull. B.O.C. 2010; 130(1): 13–32.

[54] Escalante T, Rodríguez-Tapia G, Linaje M, Illoldi-Rangel P and González-López R. Identification of areas of endemism from species distribution models: Threshold selection and nearctic mammals. TIP Rev.Esp.Cienc.Quím.Biol.2013; 16(1): 5–17.

[55] Pearson RG, Raxworthy CJ, Nakamura M and Townsend PA. Predicting species distributions from small numbers of occurrence records: a test case using cryptic geckos in Madagascar. Journal of Biogeography. 2007; 34(1): 102–117.

[56] Ruiz CJA, Durán PN, Sánchez GJJ, Ron PJ, González EDR, Medina GG et al Climatic adaptation and ecological descriptors of 42 maize races. Crop Science. 2008; 48: 1502–1512.

[57] Collins GN. The Rediscovery of Teosinte in Guatemala. Journal of Heredity. 1932; 23 (7): 261–265.

[58] Silva NCdeA, Vidal R, Costa FM, Vaio M and Ogliari JB. Presence of *Zea luxurians* (Durieu and Ascherson) Bird in Southern Brazil: Implications for the Conservation of Wild Relatives of Maize. PLoS One. 2015; 10(10):e0139034 doi: 10.1371/journal.pone.0139034 26488577 10.1371/journal.pone.0139034 PMC4619479

[59] Lumholtz C. Unknown Mexico, Vol. 1 C. New York: Scribner’s Sons 1902; p. 429.

[60] Collins GW, Kempton JH and Stadelman R. Maize investigations. Carnegie Institution of Washington, Year Book No. 36. 1937; pp. 149–150.

[61] van Devender TR. Research in the Sierra Madre Occidental of Eastern Sonora, Mexico: Grasses of the Municipio de Yécora. Arizona-Sonora Desert Museum. 2016. (https://www.desertmuseum.org/programs/yecora_grasslist.php, 30-dic-2016).

[62] Royal Gardens, Kew. Bulletin of Miscellaneous Information No. 95. 1894; pp. 373–387.

[63] National Dairy Development Board (NDDB). Nutritive value of commonly available feeds and fodder in India. Animal Nutrition Group, Anand, India. 2012; 112p.

[64] Kundu CK, Hedayetullah MD, Bera PS, Biswas T and Chatterjee S. Effect of nitrogen levels on different varieties of fodder teosinte [*Euchlaena mexicana* (L.) Schrod] in new alluvial zone of west Bengal. Forage Res. 2015; 40 (4): pp. 243–246.

[65] El-Nahrawy MA. Country Pasture/Forage Resource Profiles: EGYPT. Food and Agriculture Organization of the United Nations (FAO) 2011; 44p.

[66] Ibrahim HIM, Hassan EL-Sayed A and Eissa SMHA. Impact of Bio-Fertilization on Productivity, Grain Quality and Economic Revenue of Rayana. World Journal of Agricultural Sciences. 2015; 11 (5): 268–278.

[67] Devkota NR, Pokharel P, Paudel LN, Upreti CR and Joshi NP. Performance of teosinte (*Euchlaena mexicana*) as a promising summer-forage crop with respect to location and sowing dates considering the scenario of possible climate change in Nepal. Nepalese Journal of Agricultural Sciences. 2015; 13: 131–141.

[68] Vasey G. Grasses of the South. Department of Agriculture. Botanical Division. Bulletin No. 3. 1887; 63p.

[69] Lamson-Scribner F. Southern Forage Plants. U.S. Department of Agriculture. Farmers’ Bulletin No. 102. 1899; 48p.

[70] Hitchcock AS. Manual of the grasses of the United States. Dover Publications, Inc., 1971 New York. Second Edition of the work published in 1935.

[71] EFSA (European Food Safety Authority). Relevance of new scientific evidence on the occurrence of teosinte in maize fields in Spain and France for previous environmental risk assessment conclusions and risk management recommendations on the cultivation of maize events MON810, Bt11, 1507 and GA21. EFSA supporting publication 2016; EN-1094. 13 pp.

[72] Pardo G, Fuertes S, Fernández-Cavada S, Betrán E, Cirujeda RA, Marí LAI, et al. Presencia de teosinte (Zea spp.) como mala hierba en los regadíos del valle del Ebro. In XV Congreso de la Sociedad Española de Malherbología: La Malherbología y la transferencia tecnológica, Junta de Andalucia ed, (Sevilla, 19–22 octubre 2015), pp. 417–423.

[73] Pardo SG, Cirujeda RA, Marí LAI, Aibar LJ, Fuertes S and Taberner PA. El teosinte: descripción, situación actual en el valle del Ebro y resultados de los primeros ensayos. Vida Rural. 2016; 408: 42–48.

[74] Espinosa GFJ and Sarukhán J. Manual de malezas del Valle de Mexico. Universidad Nacional Autónoma de Mexico y Fondo de Cultura Económica, Mexico 1997; 407p.

[75] Vibrans H and Estrada JG. Annual teosinte is a common weed in the Valley of Toluca, Mexico. Maydica. 1998; 43: 45–48.

[76] Balbuena MA, Rosales RE, Valencia HJC, González HA, Pérez LDJ, Sánchez NS et al Competencia entre maíz y teocintle: efecto en el rendimiento y sus componentes. Centro Agrícola. 2011; 38(1): 5–12.

[77] Sánchez-Ken JG, Zita PGA and Mendoza CM. Catálogo de las gramíneas malezas nativas e introducidas de Mexico. Consejo Nacional Consultivo Fitosanitario, CONACOFI-SAGARPA. 2012; 433p.

[78] White RP. Cultural practices affecting maturity and yield of corn (Zea mays for whole-plant silage in short-season areas. Can. J. Plant Sci. 1978; 5g: 629–642.

[79] Kwabiah AB, MacPherson M and McKenzie DB. Corn heat unit variability and potential of corn (*Zea mays* L.) production in a cool climate ecosystem. Can. J. Plant Sci. 2003; 83(4): 689–698.

[80] Thullier W, Lafourcade B, Engler R and Araujo M. BIOMOD a platform for ensemble forecasting of species distributions. *Ecography*. 2009; vol. 32, pp. 369–373.

[81] Maxted N and Kell SP. Establishment of a global network for the *in situ* conservation of crop wild relatives: status and needs. FAO Commission on Genetic Resources for Food and Agriculture, Rome, Italy 2009; 266 p.

[82] Castañeda-Álvarez NP, Khoury CK, Achicanoy HA, Bernau V, Dempewolf H, Eastwood RJ et al Global conservation priorities for crop wild relatives. Nature Plants. 2016; 2, 16022 doi: 10.1038/nplants.2016.22 27249561 10.1038/nplants.2016.22

[83] Ramírez-Villegas J, Khoury C, Jarvis A, Debouck DG and Guarino L. A Gap analysis methodology for collecting crop genepools: A case study with *Phaseolus* beans. Plos One. 2010; 5(10): e13497 doi: 10.1371/journal.pone.0013497 20976009 10.1371/journal.pone.0013497 PMC2958131

[84] Wilkes GH. Urgent notice to all maize researchers: disappearance and extinction of the last wild teosinte population is more than half completed. A proposal for teosinte evolution and conservation *in situ*: the Balsas, Guerrero, Mexico. Maydica. 2007; 52: 49–70.

