# Supplementary figures and images for: Ecogeography of teosinte
Source: PLoS One. 2018 Feb 16;13(2):e0192676. doi: 10.1371/journal.pone.0192676 (PMC5815594; doi:10.1371/journal.pone.0192676)

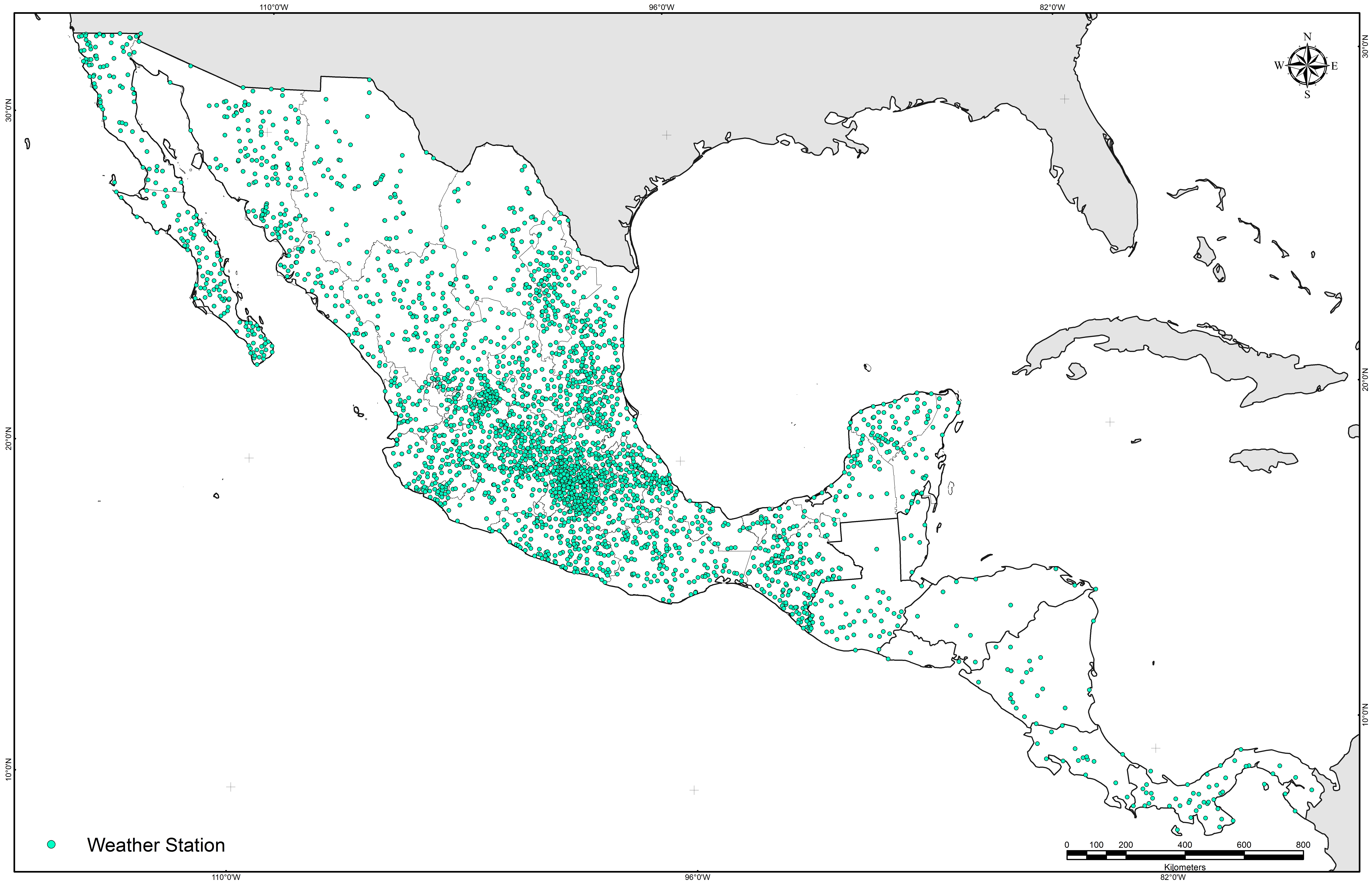

Supplement: S1 Fig — (TIF) [file pone.0192676.s001.tif]

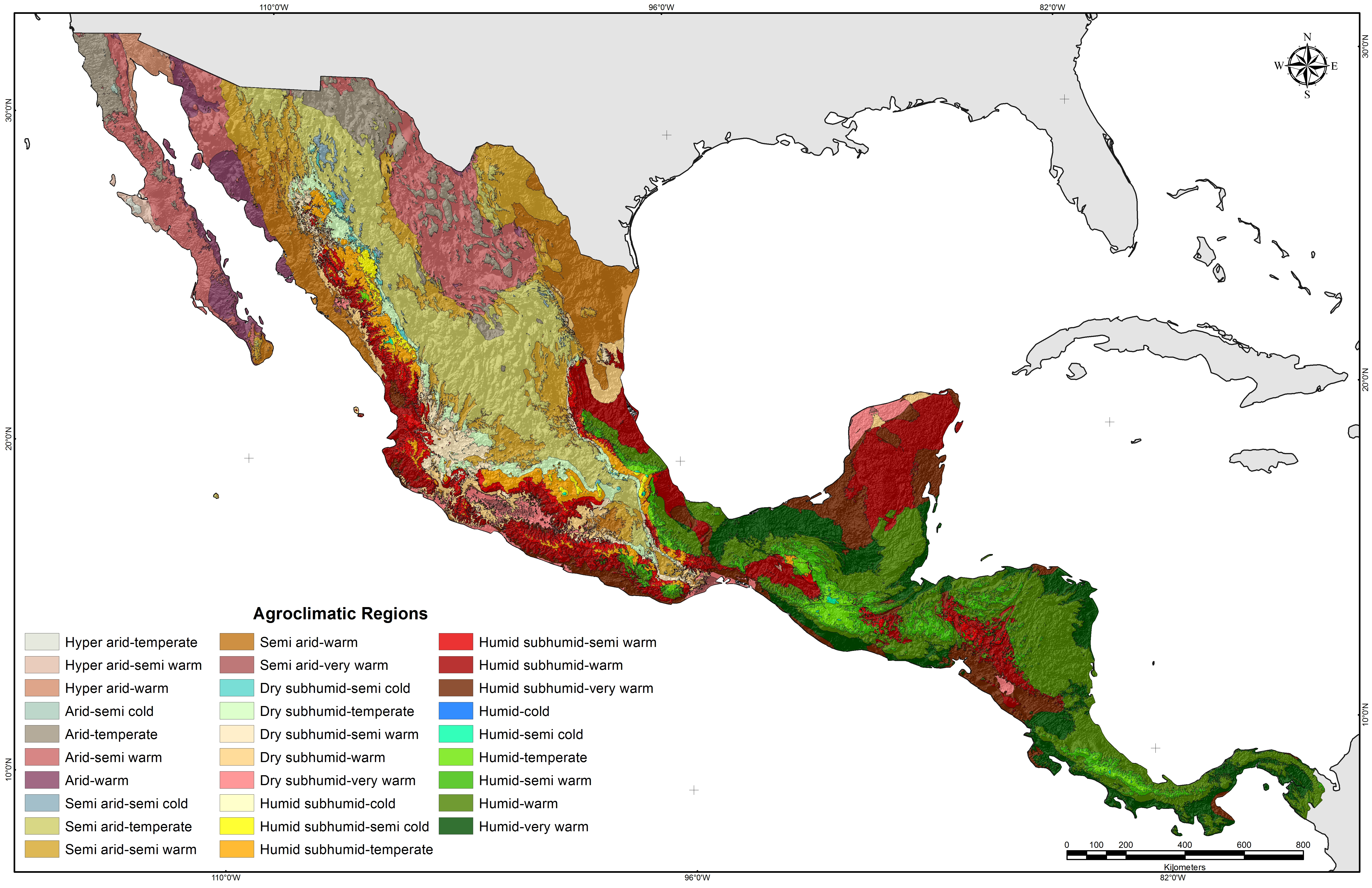

Supplement: S2 Fig — (TIF) [file pone.0192676.s002.tif]
